# Using road class as a replacement for predicted motorized traffic flow in spatial network models of cycling

**DOI:** 10.1038/s41598-019-55669-8

**Published:** 2019-12-23

**Authors:** Eric Yin Cheung Chan, Crispin H. V. Cooper

**Affiliations:** 1 Department of Geography and Planning Cardiff University, King Edward VII Avenue, Cardiff, CF10 3WA United Kingdom; 2 Sustainable Places Research Institute, Cardiff University, 33 Park Place, Cardiff, CF10 3BA United Kingdom

**Keywords:** Environmental social sciences, Energy and society

## Abstract

Recent years have seen renewed policy interest in urban cycling due to the negative impacts of motorized traffic, obesity and emissions. Simulating bicycle mode share and flows can help decide where to build new infrastructure for maximum impact, though modelling budgets are limited. The four step model used for vehicles is not typically used for this task as, aside from the expense of use, it is designed around too-large zone sizes and a simplified network. Alternative approaches are based on aggregate statistics or spatial network analysis, the latter being necessary to create a model sufficiently sensitive to infrastructure location, although still requiring considerable modelling effort due to the need to simulate motor vehicle flows in order to account for the effect of motorized traffic in disincentivising cycling. The model presented uses an existing spatial network analysis methodology on an unsimplified network, but simplifies the analysis by substituting explicit prediction of motorized traffic flow with an alternative based on road classification. The method offers a large reduction in modelling effort, but nonetheless gives model correlation with actual cycling flows (R^2^ = 0.85) broadly comparable to a previous model with motorized traffic fully simulated (R^2^ = 0.78).

## Introduction

Recent years have seen renewed policy interest in urban cycling due to increasing realisation of the negative impacts of motor traffic, obesity and emissions^1^. Some cities which are well known for their cycling infrastructures, such as Amsterdam and Copenhagen have been leading the world in terms of cycling level with 40% of trips completed by cycling^2^. Meanwhile, others such as London, New York City and Paris are investing in infrastructure or adopting pro-cycling policies^3,4^. However, with limited resources, it is crucial to assure the money is well spent. Thus, a common question to be asked when urban planners are attempting to build a bicycle-friendly environment is: where to implement cycling infrastructure for maximum effect? The economic argument is often the most persuasive to policymakers, and is underpinned by the switch of transport mode from motor vehicle to bicycle: fit people save health services money. Simulation of cyclist mode share is thus of great importance.

Aggregate statistical approaches based on spatial factors and demographics have been successful at predicting overall levels of cycling^5–9^. Another possibility is to model potential rather than predictions, where potential is defined as current travel demand over distances short enough to be cycled, whether or not such demand is currently fulfilled by cycling^10^. These models are valuable for identifying potential at coarse spatial level but once that has been established, a different model is needed to predict the effect of spatially detailed infrastructure changes. Any such model will necessarily need to determine whether a proposed infrastructure change actually lies on a route that, post-change, will actually be used, hence models must incorporate cyclist route choice^11–14^.

Motorized transport is typically simulated by the four-step model^15^: trip generation, trip distribution, mode choice and route choice. Ref. ^16^ outlines reasons why this approach has not simply been extended to active travel modelling. Most crucial from a cycling perspective is that practical deployments of the four-step model are typically (i) geared towards use on a simplified road network, and (ii) use a zonal approach when predicting trips (i.e. from residential zones to business zones). The simplified network arises because accurate vehicle modelling requires iterative assignment to determine the equilibrium state in presence of congestion, as well as junction timing models, both of which complicate analysis, so it is beneficial to simplify road networks by removing minor streets which play little role in actual motorized flow patterns. The zonal approach arises because demographic data is usually only available at zonal level. In modelling cycling, however, the zonal approach misses detailed consideration of trips that fall within a single zone, along with minor roads which may be preferred by cyclists. A further limitation of the four step model is exclusion of long terms effects of changing accessibility on land use: such feedbacks are of importance to active travel models, e.g. in residential location self-selection^16^. Finally, the budget for modelling cycling is typically much smaller than that available for motorized traffic models.

To address these issues, ref. ^17^ simplified the route choice model of ref. ^11^ and combined it with spatial network analysis to model cyclist flows, risk and mode share. This model made the simplifying assumption that cyclists travel from everywhere to everywhere subject to a maximum trip distance. Later work^18^ managed to discard these assumptions, in their place incorporating agglomeration effects, multiple trip purposes, heterogeneous preferences of different classes of cyclist, and the deterring effects of traffic and slope on mode share, to obtain a cross-validated fit with coefficient of determination R^2^ = 0.78 between modelled and measured cyclist flows. In the latter model, both mode and route choice are based on “cyclist-adjusted distance” i.e. distance with penalties applied for slope, turns, and level of predicted motorized traffic flow on each individual link within the network. Similar models of the pedestrian mode have also been produced^19^.

An ongoing weakness of these cycling models, however, is the necessity of simulating levels of vehicle traffic in order to predict its deterrent effect on cyclists. For this, a second spatial network model is used, necessarily targeted at wider spatial scale to incorporate longer vehicle trips. It is equally detailed as the cycling model, but takes a simpler approach, being in contrast to ref. ^18^, univariate, single purpose and ignoring distance decay. Nonetheless, the vehicle sub-model typically considers trips of up to 30 km from the city centre, i.e. within a circle of area 2,800 km^2^. The cycling model, by comparison, might be around 7 km in radius hence covering a circle of 150 km^2^. The vehicle model, therefore, requires data acquisition, cleaning, computation, fitting and checking of an area up to 20x greater than the cyclist model. With cycling infrastructure being planned on limited budgets it would be of great advantage to remove the requirement of a vehicle model, hence this is the contribution of the current paper, which presents an alternative formulation based on road class – an approach which has already shown promise in other cycling studies^14,20^. Road class refers to the categorisation of different roads according to their function, hierarchy, types, physical attributes etc^21^. In the current context, road class is taken to represent cyclists’ perceptions of different roads, based on behavioural expectations, motor vehicle traffic, road function, number of lanes and speed limit, the latter being indirectly related to the road capacity. Our contribution is to combine road class as a predictor of cyclist behaviour, with a spatial network analysis approach, to model cyclist flows and mode share, and compare results with existing models based on a vehicle traffic sub-model^17,18^. Results show comparable performance albeit with substantially reduced modelling effort.

## Results

Our best model, model 3, achieves cross-validated R^2^ with measured cycle flows of 0.854 and mean GEH of 1.92 (see Section 4.3 for the definition of GEH). It also achieves a cross-validated fit of R^2^ = 0.45 against census output area-level mode share data. Model 3, therefore, offers an improvement on the performance of ref. ^18^ which achieved R^2^ = 0.78 in the prediction of measured flows, and equals that study in the prediction of mode share.

A comparison of work required for the different modelling processes is given in Table 1. Note that as Cardiff is a coastal city, this may underestimate the efforts of regional models in inland cities from which hinterland extends in all directions. The modelling areas, for example, differ only by a factor of 7 in this study; and the number of network links differ by a factor of 3 as it is the less dense areas which have been excluded from the simpler model (this may not be the case in other applications e.g. modelling the centre of a large city).

**Table 1:** Comparison of modelling effort and resources for road class versus spatial network based model.

Modelling Approach	Spatial Network predicted motorized flow + Spatial Network predicted cyclist flow	Road Class predicted motorized flow + Spatial Network predicted cyclist flow
Area	1800 km^2^	242 km^2^
Links in network	74,988	23,269
Network length	1,809 km	7,646 km
Local Authorities	10	2
OpenStreetMap source data size (as shapefile)	77 MB	28 MB
Light manual checks: bridges	497 (355 motorway/primary/trunk bridges in city and region; 142 additional bridges in city)	250 bridges in city
Extensive manual checks: one-way links	Approx. 10 roads in Cardiff city comprising 113 km/2672 links	0
Links needing manual classification	0	2 roads comprising 15 km/144 links in total
Compute time Intel i7-4810-MQ, 4 cores, 2.8 GHz, 32GB. (Times are for full betweenness computations; can be reduced by sampling approximation)	~16 hours for Angular Betweenness, regional, 10, 15, 20, 25, 30, 35 km Plus 12 minutes to 10 hours depending on city cyclist model chosen (see cell to right →)	Models 1,2: ~12 minutes for 6 km roundtrip cyclist betweenness at city scale Model 3 repeatedly uses ~1.1 hours for cyclist betweenness, city, 3, 5, 8, 11, 15, 20 km round trip. In the current study repeated for 9 combinations of confidence and trip purpose (total ~10 hours); other applications may require less
Model re-runs	1 (link erroneously included in motorized model caused serious errors)	1 (reclassification of residential/non-residential dual carriageways as described in text)
Essential data for replication elsewhere	Spatial network (city and region) including one-way links	Spatial network (city) Road class
Recommended recalibration and data for replication elsewhere	Calibrate against cyclist counts or journey to work mode share Optional calibration against motorized counts	Calibrate against cyclist counts or journey to work mode share Optional verification of distance multiplier per road class vs motorized counts

Modelling effort is also contingent on the accuracy of spatial models required in each case. At the time of the study, the OpenStreetMap data often contained topology errors where links would touch or intersect at places other than endpoints, and misclassifications of one-way links. For the spatial network model of motorized flow, it was essential to manually check one-way links, as errors in their encoding could result in e.g. all motor traffic being assigned to one side of a dual carriageway only, causing the empty side to appear attractive for cycling when this is not reflected in real-world conditions. Assignment of road classes, by contrast, was mostly automatic, requiring manual intervention in only 2 cases. Topology errors in both models were fixed automatically by planarization and automatic splitting of lines at intersections. The exceptions are bridges and tunnels (‘brunels’) which were removed from the data before automatic splitting, but required manual checks at key locations to ensure correct recombination afterwards. This was needed for a larger number of cases in the motorized flow model.

The remainder of this section discusses models 1 and 2, used as stepping stones to achieve the better model 3, and a test of the effectiveness of road class as a predictor of motorized flow.

**Figure 1:**
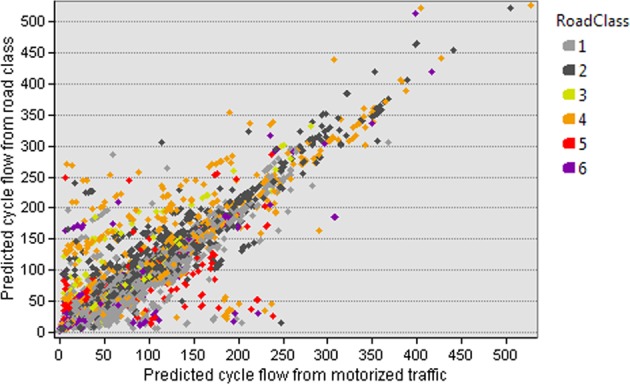
Scatter plot of predicted cycle flows on individual links from the Road Class model (model 1) vs simulated motor traffic based model^17^.

**Figure 2:**
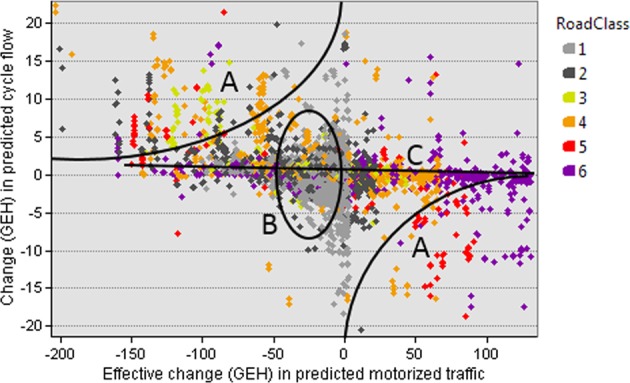
Scatter plot showing the effect of changes between ref. ^17^ and Road Class model 1. X-axis shows effective changes in predicted motorized traffic caused by substituting predicted motorized flows with road class information. Y-axis shows resulting changes in predicted cycle flow. Following ref. ^38^ differences between modelled flows x and y are expressed as GEH = $\sqrt{2{(x-y)}^{2}/(x+y)}$ albeit with sign defined to show the direction of change. See section 3 for a discussion of regions A, B, C.

Model 1 is the initial attempt to use road class to predict cycling, and used for calibration purposes only, achieving R^2^ = 0.505 in univariate fit against actual cyclist flow data, an improvement on the simulated motor flow based model of ref. ^17^ which achieved R^2^ = 0.49. Figure 1 uses a scatter plot to show the differences in prediction between model 1 and ref. ^17^. Some modelled cyclist flow has been displaced from road classes 5 to 4, reflecting model 1’s disincentivization of travelling on higher road classes, regardless of actual motorized flow. Contrary to this, other cyclist flows appear to be displaced from class 1 to 2. This is likely because replacing the predicted motorized flow of the class with its median value reduces the deterrent effect of both predicted and actual motorized flow outliers in class 2 (visible in Fig. 5). Such outliers manifest in popular parlance as ‘rat runs’: local and tertiary roads which are more popular for motorized traffic than their categorization would suggest. Unfortunately traffic count data is not available to verify this hypothesis, however, the fact that we have achieved an increase in model performance despite ignoring potentially increased actual traffic flow on ‘rat runs’ suggests a number of possibilities. Firstly it is possible that the effect is insubstantial compared to improvements in motorized flow predictions elsewhere. Secondly, it is possible that in the case of the current study area, cyclists tend to use such routes in spite of their motorized flow, perhaps because dedicated cycle lanes exist, or because the motorized flow is naturally of low speed, or managed by speed limits and traffic calming measures. Finally, it is possible that such routes entail poor cycling conditions, but no better alternatives exist. Determination of which of these is the case is beyond the scope of the current study. Figure 2 explores the difference between models in greater detail, by examining how changes in the prediction of motorized traffic affect changes of predictions in cycle traffic. Zone B contains the ‘rat runs’ discussed above: class 1 and 2 roads which, when we replace predicted motorized flow with road class information, are effectively subject to a substantial reduction in modelled motor traffic, yet exhibit little to no change in predicted cyclist flow. Not only the ‘rat runs’, but in fact, the majority of links show only a weak correspondence between the reduction of simulated motor traffic and increase of simulated cyclist flow. This is illustrated by the trend line marked C, with the exceptions being shown in the zones marked A. The reason for this seeming lack of sensitivity to predicted motorized flow is that the choice set of sensible routes through a network is naturally limited to a small number for any given trip; thus, there is scope for considerable change in the modelled cost of the alternative routes, before the cyclist’s modelled choice of route changes at all. For the modeller, this is convenient, as the lack of sensitivity (within a reasonable range) of route choice to actual motorized flow helps with our aim of discarding it from the model in favour of road class information.

Model 2 optimizes the fit against measured cycle flows by manual modification of distance multipliers to correct systematic over/under-prediction of measured flows in each road class (see Section 4.4), improving the univariate fit slightly to 0.514. Table 2 shows distance multipliers for models 1 and 2; in particular, an improved fit was achieved by increasing the distance penalty for higher road classes, in particular for class 6, non-residential dual carriageways. Model 3 (discussed at the start of this section) applies these distance multipliers in a multivariate model to achieve optimal performance with weighting λ (explained in section 4.3) equal to 0.5.

**Table 2:** Multiplicative effect on distance by motor vehicle Annual Average Daily Traffic (AADT); (i) for t = 0.04 as per ref. ^17^; (ii) calibrated to fit data in the current study.

Road Class	AADT	Distance multiplier based on Eq. (11) (model 1)	Alternative distance multiplier replacing Eq. (11) (models 2/3)
6	8698	1.4069	1.67
5	4385	1.1840	1.18
4	2253	1.0872	1.14
3	1108	1.0385	1.06
2	267	1.0042	1.01
1	13.5	0.9941	0.9943
0	0	0.9935	0.9935

Lastly, we examine the question of whether road class works for cyclist predictions by virtue of proxying actual motorized flow, by comparing spatial network^17^ and road class models for prediction of motorized traffic in Table 3. For the points where vehicle counts were conducted, the road class itself outperforms the simplified spatial network analysis used in that paper as a predictor of actual motorized flow, even taking into account the increased number of parameters (e.g. the sample mean for each road class being used as a parameter in a “model” where all roads are assigned predicted motorized flow based solely on their class). Thus we must consider in discussion the extent to which road class data may simply be a proxy for actual motorized flow.

**Table 3:** Comparison of models for predicting motorized vehicle (not cyclist) flow. We exclude traffic-free paths and include motorways to give a total of n = 107 data points for this test only. To match methodology of ref. ^17^, counts and predictions are Box Cox transformed prior to predicting R^2^ and Akaike Information Criterion (AIC), but GEH is computed on raw traffic counts. See section 4.3 for the definition of GEH.

Motorized traffic predictor	R^2^	#parameters	AIC	GEH mean
Road Class	0.87	7	1930	13.1
Simulated motor traffic as per ref. ^17^	0.84	1	1940	14.9

## Discussion

This paper has attempted to improve the transferability of spatial network analysis based cycling transport models by eliminating dependence on a detailed motor vehicle model. We have shown that replacing detailed motorized traffic flow simulation with road class information provides broadly comparable performance – in fact slightly improving on existing literature in the current case. At first glance this is surprising as we have discarded substantial information, however, several factors serve by way of explanation. Firstly, at the points for which we have motor vehicle information, the defined road class system outperforms the simplified road traffic model used in previous methods as a predictor of motorized traffic flow. Secondly, it is likely that cyclists’ perceptions of difficulty are influenced by aspects of road class beyond actual motorized flow; for example, road class proxies speed information i.e. lower road classes will carry slower moving traffic which is potentially of lesser danger to the cyclist and thus preferred, actual motorized flow notwithstanding. Although we cannot fully disentangle the influence of motorized flow versus road class in this study, the fact that model 1 (based directly on predictions of mean motorized flow for each road class) is slightly outperformed by model 2 (based on further calibration of road classes, in particular increasing the deterrence of all higher road classes except residential dual carriageways) suggests that both factors make a contribution. Thirdly, we note that the realistic option set for route choice between any two points is normally limited, therefore quite wide variance between different models in deterrence caused by motor vehicle traffic *for the same link* will often lead to the same ultimate choice of route for the cyclist, provided the modelled deterrence of each link is within sensible limits. (This should not be confused with the importance of simulating a variety of aversions to motor traffic *among cyclists*, as shown to be beneficial both by the current paper and ref. ^18^).

The performance gain shown here, although gratifying, is of an order of magnitude which could easily be outweighed by variance in results between different data sets covering different urban areas, when the model is applied elsewhere. A limitation of the study is its restriction to a single city-scale model, rather than a study of multiple regions. We therefore see our key contribution, not as an increase in modelling accuracy, but a decrease in modelling complexity through ditching the requirement for an explicit vehicle model. In the current case, the reduction in modelling effort is substantial; theoretically, the reduction could be very high indeed, e.g. if modelling a small area within a large and dense urban metropolis. This contributes to cycle infrastructure planning by making it easier to apply the spatial network model in new locations.

Should the reason for the success of road class in cycle models be due in large part to its proxying of actual motorized flow, a further limitation materializes, namely that the model should be used with extreme caution when predicting the effect of road reclassification. In these cases, verification that post-intervention road classes will continue to approximately reflect actual motorized flow is essential. However, this is likely an unusual modelling scenario (except in the case of reclassifying to prohibit motorized traffic, in which case zero motorized flow can be assumed and this limitation does not apply). The primary envisaged use of the model is in predicting cyclist flows and mode choice, possibly in the presence of new cycling links and motorized traffic prohibitions, based on an assumption that existing motorized flows remain approximately the same except in locations where prohibitions are introduced.

In reapplication of either model to new areas, recalibration of factors (road traffic deterrence or road class deterrence) against actual cyclist flow and/or area mode share is strongly recommended. This is especially the case in international use: although similar systems of road classification are widespread globally, there are substantial differences in local context. These include, for example, (1) the difference between European-style compact cities versus American-style car-oriented cities with large suburbs; (2) the difference between planned grids of regular blocks versus organically grown spatial layouts; (3) cultural differences in how cycling is perceived as a mode of transport, awareness and willingness of drivers to afford road space to cyclists. While there is reason to believe that road class remains a useful predictor of cyclist behaviour in these contexts, it is also possible that the distance multipliers applicable in different countries will differ substantially. The road class model will require verification and possibly adaptation to ensure that the classes used make sense locally: suitability of any road class system will ultimately remain unknown until a model is attempted, but local knowledge on cyclist behaviour will likely be a good predictor of the suitability of the model. Although ref. ^17^’s model based on motorized flow offers in principle a universal standard for international comparison, the cultural differences noted above still mean that the same level of flow can have different effects on behaviour depending on local context, so neither model can be used without appropriate consideration.

Optionally, motorized traffic data can be used as a starting point for road class deterrence factors as in the current study, but in the presence of cyclist data, this may not be necessary (the same can be said for calibration of the more complex motorized spatial network model for which we propose replacement).

The future likely holds numerous potential improvements for models of cycling flow, from better calibration techniques to inclusion of additional factors such as the “safety in numbers” phenomenon^22^, and combination of socio-economic with spatial network models^23^ in particular to reflect well-known class and gender imbalances in cycling^13^.

## Methodology

### Study area

Cardiff, Wales is selected as the study area for this paper. Cardiff’s existing traffic-free cycle network is quite fragmented with only the Taff Trail, a flagship cycle route which connects north and south, acting as a backbone. According to the 2011 Census of England and Wales^24^, 3.6% of working residents cycle to work in Cardiff, which is leading in Wales and higher than the average of England and Wales. Yet, there is a huge gap between Cardiff and the 10 UK cities exhibiting the highest levels of cycling to work. Cardiff Cycling Strategy 2016-2026^25^ observes that 52% of car trips in Cardiff are under 5 km and 28% of residents do not cycle now but aspire to in future, revealing large potential for increasing the cycling level. However, annual capital expenditure on cycling infrastructure by Cardiff Council and external funding combined is only £4 per resident, a low investment compared to internationally renowned cycling cities Amsterdam and Copenhagen which invest around £18 per resident. A larger investment in expanding the cycle network is expected to assist in realizing this potential.

### Data

This paper is based on a spatial network provided by OpenStreetMap (OSM), a public and crowd sourced mapping system^26^. In terms of cycle network coverage, continuity, attributes and recency, ref. ^27^ found OSM to be a better mapping system than Ordnance Survey (OS). Slope data for the spatial network is taken from Ordnance Survey Terrain 50; this misses small scale changes in height such as those encountered on bridges/underpasses, however, captures most terrain effects and has the advantage of being free to use under and OpenData license.

To calibrate the models, two sources of actual cycle flow data were used. The Department for Transport estimate, by combination of manual and automatic survey and interpolation^28^, the annual average daily traffic (AADT) of both motor vehicles and pedal cycles at 107 on-road locations in Cardiff. This is supplemented by cycle flow data from 14 traffic-free locations collected by electronic sensors belonging to Cardiff Council. As both sources used different methodologies to collect cycle flow data, they are not directly comparable, in particular due to the Department for Transport not taking localized weather conditions into account when surveying cycling behaviour. However, both sources are important to the calibration process and thus must be combined. We follow ref. ^17^ in using a dummy variable to account for data source in the final predicted flow model.

The motor vehicle flow predictions in Cardiff are obtained from the motor vehicle flow sub-model in ref. ^17^, which has a good correlation (R^2^ = 0.84) with measured vehicle flows.

Mode share data is taken from a total of 1077 census Output Areas (Office for National Statistics, 2011).

### Network analysis

This paper applies the publicly available Spatial Design Network Analysis + (sDNA+) toolkit in ArcGIS^29^. To calibrate the effect of road class in our models 1 and 2, we make use of the simpler models presented in ref. ^17^, and to obtain our final results we add in model 3 the extensions of multiple trip purpose, distance decay, heterogeneous cyclist ability and agglomeration detailed in ref. ^18^. The remainder of this section summarizes the models in these two papers.

Both of these models make use of spatial network betweenness^30^ for predicting flows. Intuitively this can be conceived as simulating the shortest trips from everywhere to everywhere, subject to a definition of distance which reflects cyclist preferences, and a maximum distance for the trip. Although apparently indiscriminate in handling of origins and destinations, the correlation of network density with jobs and homes^31^ has the effect that denser areas are modelled as generating more trips. The betweenness approach thus has a history of providing a reasonable fit to vehicle^32,33^ and pedestrian^34^ data. The formula used for betweenness is

1 $$Betweenness(x,rmin,rmax,{d}_{routing},{d}_{radius})=\sum _{y\in N}\,\sum _{z\in R(y,rmin,rmax,{d}_{radius})}OD(y,z,x,{d}_{routing})W(z)$$

2 $$OD(y,z,x,{d}_{routing})=\{\begin{array}{ll}1 & if\,x\,is\,on\,the\,shortest\,path\,from\,y\,to\,z\,as\,defined\,by\,metric\,{d}_{routing}\\ 1/2 & if\,x=y\ne z\,or\,x=z\ne y\\ 1/3 & if\,x=y=z\\ 0 & otherwise\end{array}$$

where x, y and z are links in the network N, and *R*(*y*,*rmin*,*rmax*,*d*_*radius*_) is the subset of the network closer to link y than a threshold radius *rmax* but further from y than *rmin*, according to the distance metric *d*_*radius*_. The OD(y,z,x,d) function defined in Eq. (2) describes the proportion of link x that falls on the shortest path from the middle of link y to the middle of link z, with partial contributions for links which form the endpoints of the shortest path^18^. This is equivalent to the original definition of betweenness^30^ under the assumption that shortest paths are unique, and subject to adaptation for spatial network representation in which, under dual representation^35^, links are considered as nodes and – as nodes representing links occupy more than a single point in space – definitions of partial contributions are required for trip endpoints. W(z) is a weighting function for the importance of destination z.

Reference ^17^ and our models 1 and 2 use network-Euclidean distance for *d*_*radius*_, set rmin = 0, rmax = 3 km, W(z)=1 and for *d*_*rauting*_ use the definition of cyclist distance outlined in Section 4.4, Eq. 9 below (a Euclidean network distance adjusted for slope or motorized traffic). Variables are normalized using a Box-Cox transform prior to regression.

Reference ^18^ and our model 3 augment the “everywhere to everywhere” assumption with a variety of different trip purposes: trips to each network link, extra trips to each link within the city centre (as defined by a threshold of urban density – this can also be interpreted as incorporating agglomeration effects), trips to recreational cycling facilities. Each of these is duplicated for cyclist classes of varying confidence i.e. varying aversion to motor traffic, and disaggregated within various distance bands (3, 5, 8, 11, 15 and 20 km round trips) to account for distance decay; in contrast to ref. ^17^ these distances are interpreted as adjusted for slope and motorized traffic because we use cyclist distance (Section 4.4 Eq. 9) for *d*_*radius*_ as well as *d*_*rauting*_. The multiple trip/cyclist combinations can also be interpreted as a simulation of non-interacting agents. In modelling terms, this means that multiple betweenness values are computed for each link, based on different values of *d*_*rauting*_, *d*_*radius*_, *rmin*,* rmax* and W(z), where

3 $$W(z)=\{\begin{array}{ll}1 & if\,z\,is\,a\,destination\,of\,interest\\ 0 & otherwise\end{array}$$

The sDNA + software automatically sets* rmin* and* rmax* given the desired distance bands above. Traffic aversion and hence *d*_*rauting*_ and *d*_*radius*_ are modified by changing the value of parameter t in Eq. (9). A betweenness value for each distance band is computed for each possible combination of t = {0.4,0.6,0.8} with W(z) representing {everywhere, city centre, recreational facilities} respectively. The multiple betweenness values are used as independent variables in a linear regression to predict cyclist flows using the sDNA Learn tool:

4 $$flow={\beta }_{0}+{\beta }_{source}\,source+{\beta }_{1}betweennes{s}_{1}+{\beta }_{2}betweennes{s}_{2}+\ldots $$

where the βs are regression coefficients, and *source* is a dummy variable set to 0 if the actual flow was recorded by the Department for Transport and 1 if recorded by Cardiff Council.

Cross-validated ridge regression is used to handle inherent collinearity and prevent overfit^36,37^; models can thus be compared using a cross-validated coefficient of determination (R^2^). The Box-Cox transform is inappropriate in a multiple regression context and is therefore replaced with a weighting scheme

5 $$RW(y)={y}^{\lambda }/y$$

Where RW(y) is the regression weight for a data point with dependent variable value y, and λ is a calibration parameter (similar to that in the Box Cox transform, and unrelated to the regularization parameter λ in ridge regression) such that regressing with λ = 1 minimizes absolute errors while λ = 0 minimized relative errors. The actual value of λ is chosen so as to minimize the GEH (Geoffrey E. Havers) error statistic popular in transport planning^38^, which captures a mixture of absolute and relative error in residuals:

6 $${\rm{GEH}}=\sqrt{2{({\rm{x}}-{\rm{y}})}^{2}/({\rm{x}}+{\rm{y}})}$$

To predict mode share, ref. ^18^ and our model 3 calibrate a multivariate model based on network reach within all the distance bands, trip purposes and for all the cyclist types outlined above, where

7 $$Reach\,(x,rmin,rmax,{d}_{radius})=\sum _{y\in R(x,rmin,rmax,{d}_{radius})}W(y)$$

8 $$journey\,to\,work\,mode\,share={\beta }_{0}+{\beta }_{1}Reac{h}_{1}+{\beta }_{2}Reac{h}_{2}+\ldots $$

where the βs are regression coefficients. As mode share data is only available on a zonal basis, the reach variables are averaged over all links within each zone to provide the independent variables for regression.

### Definition of distance

The cycling models of betweenness and network density are both based on a cycling distance metric which accounts for the effect of slope, levels of motorized traffic and straightness on the distance perceived by the cyclist. Ref. ^17^ begins with the findings of ref. ^11^, simplifying and recalibrating to arrive at the definition outlined in Eqs. (9–11):

9 $$\begin{array}{c}cyclist\,distance=Euclidean\,network\,distance\times slopefa{c}^{s}\times trafficfa{c}^{t}\\ +\,cumulative\,angular\,change\times \frac{67.2}{90}\times a\end{array}$$

where

10 $$slopefac=\begin{array}{ll}1.000 & if\,slope < 2 \% \\ 1.371 & if\,2 \%  < slope < 4 \% \\ 2.203 & if\,4 \%  < slope < 6 \% \\ 4.239 & if\,slope > 6 \% \end{array}$$

11 $$trafficfac=0.84\,{e}^{\frac{AADT}{1000}}$$

and AADT is the predicted annual average daily flow of motorized vehicles on the link. The cycling distance is measured as a round trip and it is assumed that a cyclist adopts the same route for both outward and return journey. Calibration in that paper is achieved by varying the parameters a, s and t, with the best fit on the Cardiff data set given by a = 0.2, s = 2, t = 0.04.

Motor traffic enters the definition of distance in Eq. (11). For the present study, we replace this with a *trafficfac* defined for each road class. In model 1 this is defined as per Eq. (11) albeit replacing individual simulated AADT for each link, with a length-weighted median simulated AADT for the road class within the smaller cyclist network model (i.e. excluding the larger network model used to predict motorized flow in ref. ^17^). We use these values as starting points for further optimization of the model parameters, with the endpoint of optimization being model 2. Optimization was conducted by manual adjustment of parameters to correct systematic over/underprediction of cyclist flows per road class: e.g. non-residential dual carriageways had lower actual cyclist flow than predicted, so their *trafficfac* was increased, etc. Finally, we take the *trafficfac* parameters derived in our model 2 and apply them to replace *trafficfac* in the methodology of ref. ^18^ (described in more detail in section 4.3 above), giving our best predictions of cyclist flow and mode share in model 3.

### Road categorisation

**Figure 3:**
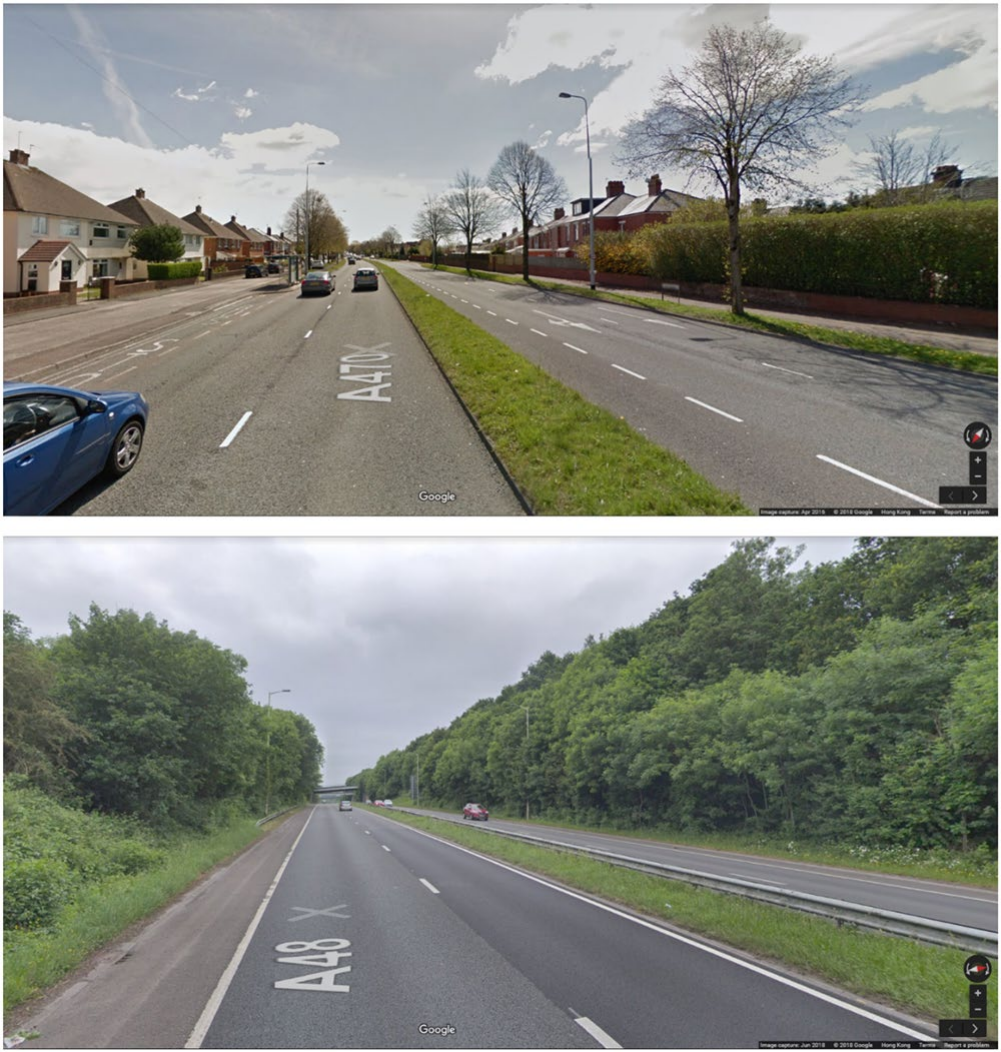
Northern Avenue residential dual carriageway (above) vs Eastern Avenue non-residential dual carriageway (below) (Map data copyright Google 2018).

The practice of road classification is pervasive in modern transport planning, and hence ubiquitous in higher income, as well as widespread in middle-income countries worldwide. The UK Department for Transport defines five types of road which are broadly comparable to those used in other countries: motorways, A roads, B roads, classified unnumbered and unclassified^39^. We reviewed these categories within the study area to determine whether we believed them to capture sufficient details of the urban environment for our purpose of replacing predicted traffic flow in the models of^17,18^. Of particular concern was that A roads in the UK can be both major and minor arterials, and separately, be built with either single or dual carriageway design. Furthermore, the cycling characteristics of dual carriageway A roads differ substantially depending on whether or not they are fronted by residential properties. Figure 3 shows an example, contrasting a residential dual carriageway bordered by pedestrian sidewalks and joined by private driveways, speed limit 40mph, with a non-residential dual carriageway which is functionally similar to a motorway with a variety of speed limits up to 70mph. To capture these differences to the cycling environment, we define three road classes extracted from A roads: residential single carriageway, residential dual carriageway and non-residential dual carriageway. The remainder of the Department for Transport’s classes were considered adequate for our purpose. Defined road classes with general definitions/functions/features and the associated conversion to UK standard are set out in Table 3. Comparison of models for predicting motorized vehicle (not cyclist) flow. We exclude traffic free paths and include motorways to give a total of n=107 data points for this test only. To match methodology of ref. ^17^, counts and predictions are Box Cox transformed prior to predicting R^2^ and Akaike Information Criterion (AIC), but GEH is computed on raw traffic counts. See section 4.3 for definition of GEH.

**Figure 4:**
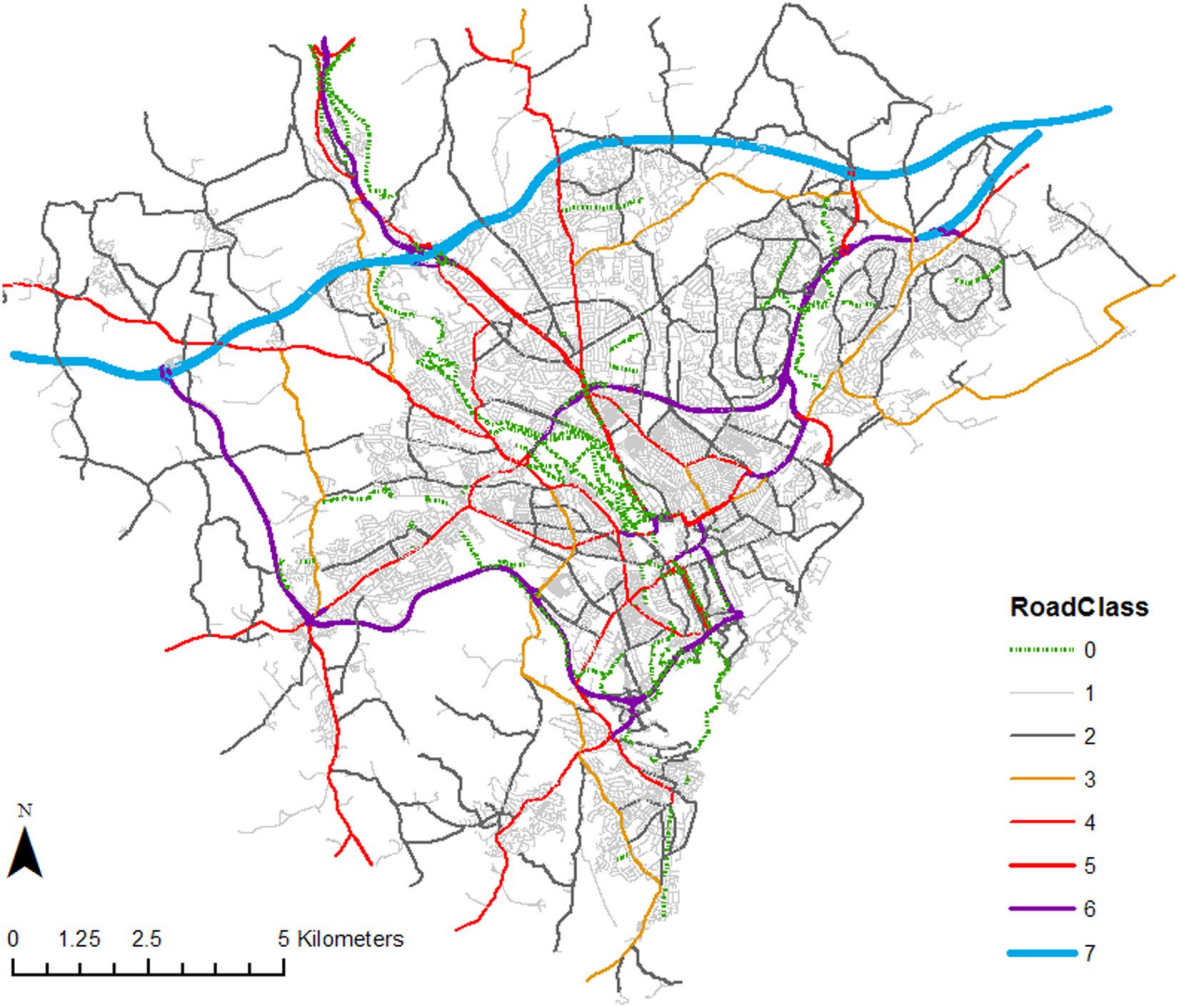
Spatial network of Cardiff with road classes defined. (Underlying spatial data copyright OpenStreetMap contributors; map produced in ArcGIS 10.3 https://www.arcgis.com).

Table 4. Having defined these road classes it is also necessary to define the mapping through which they are extracted from OSM, based on OSM’s defined highway types. Table 5 shows possible values for the ‘*highway*’ tag in OpenStreetMap. For instance, *trunk* refers to a dual carriageway A Road usually; *primary* refers to a single carriageway A Road; *secondary* refers to a B Road; and *tertiary* refers to a classified unnumbered road. In scenarios where a link is actually a single carriageway but classified as trunk or a dual carriageway, another attribute ‘*oneway*’ is used to assure single and dual carriageways are correctly differentiated. For lower level road types, information from OSM tends to be detailed and needs to be consolidated to match with the defined road classes or to be excluded when it is not relevant to cyclists. For instance, *living_street* and *residential* are both classified as local roads while *bridleway* and *track* can be excluded as they do not appear within Cardiff city limits. Table 6 shows the derivation of our road classes from OSM data and Fig. 4 the resulting road categorisation in Cardiff.

**Table 4:** Road classes defined for this paper.

Road Class	Description	General Definitions/Functions/Features	Conversion to the UK Road Classification
7	Motorways (not included in cyclist model)	Major road designated for regional connection, accommodating fast and high traffic flows. Central reservations used to safely separate high speed traffic flows. Other roads connect only at dedicated on/offramps allowing acceleration/deceleration. Cycling prohibited.	Motorways (M)
6	Non-residential Dual Carriageways	Major arterials forming a continuous route between two primary destinations. Central reservations used to safely separate high speed traffic flows. This road class locates outside residential areas with few connecting roads and no pedestrian sidewalks.	A Roads
5	Residential Dual Carriageways	Major arterials forming a continuous route between two primary destinations. Central reservations used to safely separate high traffic flows. This road class locates within residential areas with more connecting roads and pedestrian sidewalks.	A Roads
4	Primary Roads	Major arterials forming a continuous route between two primary destinations with lower capacity and speed than above-mentioned major arterials due to the design.	A Roads
3	Secondary Roads	Minor arterials which feed traffic between the major arterials and minor roads.	B Roads
2	Tertiary Roads	Minor roads which mainly collect traffic from local roads to arterials.	Classified Unnumbered
1	Local Roads	Local roads with high degree of access to residential properties and other trip endpoints.	Unclassified
0	Traffic-free Paths	Paths for use of cyclists and pedestrians only.	N/A

**Table 5:** Values of ‘highway’ tag in OpenStreetMap data used for Cardiff/

highway=	Number of features	Length (km)	Description
cycleway	277	66	Paths for cycling
footway	27	2	Footpaths
living_street	2	0	Streets where pedestrians have priority over cars
motorway	69	53	Motorways or freeways
motorway_link	25	10	Motorways or freeways
path	21	5	Unspecified paths
pedestrian	17	1	Pedestrian only streets
primary	539	128	Primary roads
primary_link	34	5	Primary roads
residential	5833	793	Roads in residential areas
road	11	2	Roads in residential areas
secondary	162	52	Secondary roads, typically regional
service	3277	336	Service roads for access to buildings, parking lots, gas station, etc.
services	1	1	Service roads for access to buildings, parking lots, gas station, etc.
steps	5	0	Flights of steps on footpaths
tertiary	654	174	Tertiary roads, typically local
tertiary_link	3	0	Tertiary roads, typically local
track	1	0	For agricultural use
trunk	175	61	Important roads; typically divided
trunk_link	57	13	Important roads; typically divided
unclassified	824	235	Smaller local roads

**Table 6:** Derivation of road classes used in the study from tags in OpenStreetMap.

Road Class	Description	Selection from the OSM
7	Motorways	highway = motorway OR highway = motorway_link
6	Non-residential Dual Carriageways	highway = trunk OR highway = trunk_link *manual classification needed
5	Residential Dual Carriageways	highway = trunk OR highway = trunk_link *manual classification needed
4	Primary Roads	highway = primary OR highway = primary_link OR (highway = trunk AND oneway = F)
3	Secondary Roads	highway = secondary OR highway = secondary_link
2	Tertiary Roads	highway = tertiary OR highway = tertiary_link
1	Local Roads	highway = living_street OR highway = residential OR highway = unclassified
0	Traffic-free Paths	highway = cycleway

**Figure 5:**
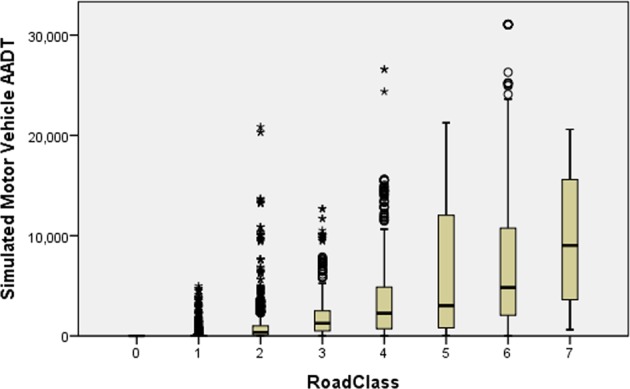
Box plot of simulated Annual Average Daily Traffic (AADT, vehicles/hour) on each link, categorized by road class. Horizontal line shows median, box shows quartiles, T bars extend 1.5x height of the box, O and * show outliers and extreme outliers respectively.

We use the vehicle sub-model of ref. ^17^ to estimate AADT on each link. As with previous literature^33,40^ this is based on angular betweenness i.e. the definition of distance is cumulative angular change, thus preferring routes with the least change of direction whether at junctions or on links. Such routes usually have priority and thus to some extent proxy shortest travel time. A range of trip distances range from 10 to 30 km are tested, picking the best fit to actual motorized flow for use in predicting AADT. Table 7 and Fig. 5 show the distribution of simulated AADT across road classes. Noting (i) the presence of AADT outliers within each road class, and (ii) that cyclists are sensitive to the distance they must travel within each traffic band, we take a length weighted median AADT for each road class as representative.

**Table 7:** Summary of distribution of simulated Annual Average Daily Traffic (AADT in vehicles/hour) across different road classes.

Road Class	Number of links	Length (km)	Mean AADT per link	Median AADT per link	Length weighted Mean	Length weighted Median
7	90	54	9352	9024	11556	9798
6	521	98	7403	4819	10377	8698
5	144	15	6358	3016	8958	4385
4	948	83	3414	2257	3762	2253
3	443	48	2296	1273	1856	1108
2	2585	280	918	368	792	267
1	18102	1208	70	15	75	13
0	526	78	0	0	0	0

## Data Availability

Measured traffic-free cycle path flows remain property of City of Cardiff Council. The remaining data is publicly available (OpenStreetMap, UK Census, Department for Transport) and the software likewise. An open source release of sDNA is now available^41^.
